# Exploring the Levels of Stress, Anxiety, Depression, Resilience, Hope, and Spiritual Well-Being Among Greek Dentistry and Nursing Students in Response to Academic Responsibilities Two Years After the COVID-19 Pandemic

**DOI:** 10.3390/healthcare13010054

**Published:** 2024-12-31

**Authors:** Polyxeni Μangoulia, Antonia Kanellopoulou, Georgia Manta, Georgios Chrysochoou, Evangelos Dimitriou, Theodora Kalogerakou, Maria Antoniadou

**Affiliations:** 1 Faculty of Nursing, School of Health Sciences, National and Kapodistrian University of Athens, 11527 Athens, Greece; gmanta@nurs.uoa.gr; 2 Department of Dentistry, School of Health Sciences, National and Kapodistrian University of Athens, 11527 Athens, Greece; akanel@dent.uoa.gr (A.K.); geochrisox@dent.uoa.gr (G.C.); dimitriou.vagos@gmail.com (E.D.); tkalogerakou@hotmail.com (T.K.); 3 Certified Systemic Analyst Program (CSAP), Systemic Management, University of Piraeus, 18435 Piraeus, Greece

**Keywords:** anxiety, stress, depression, hope, resilience, well-being, dentistry students, nursing students

## Abstract

**Background:** Dentistry and nursing students experience significant anxiety, negatively impacting their well-being and academic performance. **Objectives:** This study aims to assess the prevalence and relationships of stress, anxiety, depression, resilience, hope, and spiritual well-being among dentistry and nursing students, identify demographic influences and propose strategies to enhance resilience and well-being. **Methods:** This study surveyed 271 students attending Greece’s departments of dentistry and nursing at the National and Kapodistrian University of Athens, using an electronic questionnaire aimed to assess stress, anxiety, and depression (depression, anxiety, stress scale—DASS-21); resilience (resilience assessment questionnaire—RAQ8, brief resilience scale—BRS); hope (adult hope scale—AHS); and spiritual well-being (functional assessment of chronic illness therapy–spiritual well-being scale—FACIT-Sp-12). The survey also collected demographic data to identify factors influencing these variables. Statistical analyses, including hierarchical multiple linear regression and *t*-tests, were performed to analyze the relationships between variables. **Results:** The sample included 145 dentistry and 126 nursing students, with 68.6% female and 80.1% undergraduate. Half of the students reported mild or higher levels of stress (48.7%), anxiety (51.3%), and depression (53.5%). The prevalence of depression was the highest in our sample, followed by anxiety and stress. Higher family wealth was associated with reduced stress levels, while female undergraduate students reported higher levels of anxiety than their male counterparts. Hope was a strong predictor of resilience, but stress and worry had a negative correlation. **Conclusions:** Promoting students’ well-being and academic success requires effective stress-reduction and resilience-building techniques to improve students’ performance and support future healthcare professionals’ personal sustainability and holistic growth.

## 1. Introduction

Stress is defined by Lazarus and Folkman (1984) as “the internal and external demands that test or exceed the available response capabilities, in the individual’s estimation” [1]. It refers to the physiological response of the body to various stress factors [2]. Anxiety, on the other hand, is the feeling of fear that arises in response to threatening or stressful situations. While anxiety is a normal response to danger, persistent anxiety may indicate a disorder [2]. Anxiety disorders affect approximately one in nine people and are particularly common among dentistry and nursing students worldwide [3,4].

Resilience is maintaining stable and healthy mental and physical functioning in the face of adversity, such as separation, loss, or life-threatening situations, as defined by Bonanno (2004) [5]. According to its definition, “resilience is the ability of a person exposed to a single and very unpleasant event (separation, loss, violent life-threatening situation) to maintain relatively stable and healthy levels of mental and physical functioning, as well as the capacity for creative experiences and positive emotions.” It involves quick recovery from mental health adversity and acts as a protective factor against stress, anxiety, and depression [6,7,8,9]. Luthar (2006) and Tsigaropoulou (2020) further define resilience as “positive adaptation despite adversity” [8,10]. It represents the effort people make to recover and adapt effectively after challenging experiences [8,9].

A meta-analysis by Elani et al. (2014) showed that dentistry students experience significant anxiety throughout all years of their studies due to the demanding nature of their education [11]. Other studies also highlight the negative impact of heightened stress on students’ health and well-being [11,12,13]. Studies indicate that factors contributing to anxiety include dealing with psychiatric patients, patients with medical problems, uncooperative patients, managing a syncopal episode, tooth extraction, extracting the wrong tooth, accidental exposure to blood, soft tissue injury due to incorrect handling, administering incorrect treatment, and finally, infection from patients, which appears to be the main anxiety-inducing factor [12]. The COVID-19 pandemic heightened anxiety levels among dentistry students, particularly compared to the general population and medical students [14]. Gender differences have been noted, with female students experiencing higher anxiety levels than males [15,16]. Conflicting findings exist regarding the progression of anxiety across the years of dental education. Some studies suggest higher anxiety during preclinical years, while others show increased anxiety during clinical practice [15,16,17]. The high prevalence of stress among dentistry students emphasizes the need for support programs and preventive measures to aid those struggling with anxiety [18].

Resilience has been identified as a protective factor for dentistry students managing stress. Montas et al. (2021) found that resilience positively influences academic achievement, while factors like ethnic origin and major life events, such as the COVID-19 pandemic, affect resilience levels [12,19]. Dentistry students, however, generally show moderate resilience levels, lower than medical students, which are primarily attributed to higher levels of emotional exhaustion and reduced personal performance among dentistry students [20]. Some studies, however, report relatively high resilience among dentistry students, which is linked to factors such as gender (with females showing more resilience than males), race, and mental health [21].

Moreover, a study by Dafogianni et al. (2022) during the first wave of COVID-19 revealed that 44.8% of nursing students experienced depression, 36.8% had anxiety, and 40.3% reported stress. Female students faced higher levels of anxiety and stress compared to males, while male students demonstrated greater mental resilience [4]. Final-year students were better at developing coping strategies than first-year students [4]. Factors contributing to anxiety included demanding coursework, balancing personal life with studies, exposure to stressful situations, and making ethical decisions during clinical practice [22,23,24,25]. Thomas-Davis et al. (2020) found that anxiety affects nursing students academically, physically, and mentally [26]. The emotional and mental health of nursing students worsened during the pandemic [27], but resilience helped mitigate the negative impact of stress [28]. Studies indicate that hospital environments and lack of experience heightened anxiety in the early years of nursing education, as students faced stressful situations, conflicts with staff and patients, limited skills, and fear of mistakes [29,30,31]. Nursing students also face individual pressures such as depression, anxiety, and limited family time [32]. McCarthy et al. (2018) reported that anxiety reduces critical thinking and success and impedes decision-making [23].

Further, a positive correlation was found between resilience and performance in undergraduate nursing studies, including professional placements [33]. Resilience plays a crucial role in helping nursing students navigate adversities and stressful situations during their education [34,35]. After graduation, this resilience enables them to continue contributing effectively as Registered Nurses [34]. Smith et al. (2023) reviewed global data showing that nursing students experienced high stress levels during the COVID-19 pandemic. Resilience was widely recognized as a crucial protective factor against stress and psychological issues. Students with higher resilience were more likely to persevere in their studies despite pandemic challenges. The study emphasizes the importance of integrating resilience-building activities into undergraduate nursing programs to support academic and clinical success [35].

The existing literature identifies stress, anxiety, and resilience as critical factors for dentistry and nursing students, yet several research gaps remain. A thorough examination of the stressors peculiar to dentistry and nursing, which can differ greatly from those faced in general medical training, must be enabled [36,37]. There is a distinct absence of comparison studies [38], especially between dentistry and nursing students. Most research focuses on each group independently, overlooking the shared stressors and coping mechanisms [4,35]. Furthermore, the influence of demographic factors such as family income, region of origin, and year of study on resilience and anxiety remains underexplored, especially in non-Western context countries [19,23]. There is also a lack of longitudinal research tracking how stress and resilience evolve throughout students’ education, especially in response to global crises like the COVID-19 pandemic [11,35]. Although resilience is recognized as a protective factor, resilience-building interventions specific to healthcare students remain underdeveloped [7,10].

Stress, anxiety, and depression have an impact not only on students’ psychological well-being and academic performance but also on communication and therapeutic involvement with patients during clinical placement, as well as the quality and safety of healthcare delivery [39]. Indeed, student stress and worry can affect patients by impairing motivation, communication, learning, clinical practice, and overall academic performance [40,41,42,43]. The well-being of healthcare workers is critical to individual health and the healthcare system’s safety, efficacy, and efficiency [44]. Furthermore, for students to continue pursuing their careers with a sense of contentment, educators must better grasp the obstacles they will face as they transition from their undergraduate degrees to employment [45]. Stressors and elements that promote resilience must be addressed to improve nursing and dentistry students’ retention and job satisfaction.

As revealed from the relevant literature, the resilience and well-being of dentistry and nursing students are paramount in enhancing sustainability for future healthcare professionals in the two fields. Therefore, in this study, we aim to examine the levels of stress, anxiety, depression, resilience, hope, and spiritual well-being among dentistry and nursing students at the Department of Dentistry and Nursing of the School of Health Sciences of the National and Kapodistrian University of Athens during their undergraduate and postgraduate studies and to investigate the factors contributing to anxiety and stress among them. To collect data, we employed several validated measurement tools to capture these psychological dimensions: the Depression, Anxiety, and Stress Scale (DASS-21) to assess mental health symptoms, the Functional Assessment of Chronic Illness Therapy–Spiritual Well-Being Scale (FACIT-Sp-12) to measure spiritual well-being, the Adult Hope Scale (AHS) to measure levels of hope, and two resilience-focused tools—the Resilience Assessment Questionnaire (RAQ8) and the Brief Resilience Scale (BRS) [46,47,48,49,50,51,52]. Each tool was chosen for its reliability and validation within Greek populations, allowing a comprehensive evaluation of the students’ stress, anxiety, resilience, hope, and spiritual well-being. Conducted at the National and Kapodistrian University of Athens, this study also considers the unique academic pressures faced by Greek dentistry and nursing students. These students are introduced to a complex educational landscape shaped by high clinical and academic demands, which were intensified by the COVID-19 pandemic [46]. We further aim to evaluate how factors such as gender, year of study, family income, and region of origin may influence anxiety, resilience, hope levels, and spiritual well-being within these student populations.

## 2. Materials and Methods

This study follows a cross-sectional design chosen to collect data at one point in time and to analyze relationships between variables. Data were collected using a self-administered questionnaire. The study design is based on similar studies that have been identified in the field [46]. Further, this study was performed in two stages. A literature review was conducted, and the initial questionnaire was formulated. This was followed by piloting the questionnaire with 10 students from each department who voluntarily agreed to complete it. Factors not addressed during this stage were excluded from the final questionnaire. The final questionnaire of the study underwent one review by a team of four professors specialized in studies of this nature (two from each department). The finalized questionnaire was uploaded to Google Forms and distributed to students of both departments. The lead researcher of each department was responsible for sending the online questionnaire link to the ethics committee of each department to obtain the necessary approval and ensure that all students in each department would have the opportunity to participate in the study in the same manner. The questionnaire was anonymous, and no personal data were collected. Participation was voluntary, and no form of compensation was provided for participation. Each student should complete the questionnaire ONLY once. The questionnaire was open for two months, during March and April 2024, and the participation information bulletin with instructions and the link was sent four times (every 15 days) by the secretariat of each department.

Participants included in this study were undergraduate and postgraduate students enrolled in the nursing and dentistry programs, aged 18–30, who were able to provide informed consent. Undergraduate students pursue their first academic degree, typically a bachelor’s, and build foundational knowledge. Postgraduate students have already earned an undergraduate degree and are advancing their education through specialized master’s programs. We excluded participants who had a history of psychiatric conditions, were taking medications that could influence cognitive or emotional performance (including medication for anxiety), or did not meet the inclusion age or program criteria.

To determine the appropriate sample size, a power analysis was conducted with an expected power of 0.80 and a significance level of 0.05. Based on these parameters, this study required a minimum sample size to detect meaningful differences across the measured variables (stress, anxiety, resilience, hope, and spiritual well-being). The final sample of 271 participants, which included both dentistry and nursing students, met this requirement, providing adequate statistical power to identify significant differences and relationships within the data.

### 2.1. Designing the Study Questionnaire

#### 2.1.1. Methodology for Questionnaire Design

The instrument was constructed by packaging several validated tools. We used the following questions based on a literature review regarding anxiety and resilience and questions aiming to address anxiety and enhance resilience (Appendix A and Appendix B): depression, anxiety, and stress scale (DASS-21) [47]; the functional assessment of chronic illness therapy–spiritual well-being scale (FACIT-Sp-12) [48]; the adult hope scale (AHS) [49]; the resilience assessment questionnaire (RAQ8) [50]; the brief resilience scale (BRS) [51]. The DASS-21 (depression, anxiety, stress) scale has been validated for the Greek population by Lyrakos et al. (2011) [52]. For the depression subscale, Cronbach’s a was 0.83; for the anxiety subscale, Cronbach’s a was 0.81; and for the stress subscale, Cronbach’s a was 0.89. All rates were satisfied. The adult hope scale by Snyder et al. (1991) [49] has been examined for reliability and validity. The functional assessment of chronic illness therapy–spiritual well-being 12 (FACIT-Sp-12) scale–non-illness was evaluated and rated for reliability and validity in the Greek language by Fradelos et al. (2016) [53] with satisfied internal consistency (Cronbach’s a = 0.789). The resilience scale (BRS) is validated for the Greek population by Kyriazos et al. (2018) [54], with Cronbach’s a = 0.80–0.91 as satisfied. The resilience assessment questionnaire by Mowbray (2011), which we utilized in the present study, is a reliable and validated tool, too [50]. The reliability and validity of these tools have been analyzed in Appendix B.

The questionnaire consisted of 5 distinct sections: (1) Section 1 included questions related to demographic characteristics, (2) Section 2 included questions comprising the DASS-21 and FACIT-Sp-12 scales, (3) Section 3 included questions comprising the Adult Hope Scale (AHS), (4) Section 4 included questions comprising the Resilience Assessment Questionnaire (RAQ8), (5) Section 5 included questions comprising the Brief Resilience Scale (BRS). The sections are analyzed in Appendix B.

#### 2.1.2. Translation of the Research Tools

The translation of the questionnaires from the original language to Greek followed a structured process to maintain both conceptual and linguistic equivalence. We employed the widely accepted forward-translation and back-translation methods to ensure the translated version retained the original meaning and cultural relevance [55]. For the forward translation, two independent bilingual translators with expertise in the field translated the questionnaires from the original language into Greek. For the back translation: A third independent bilingual translator, who had no prior knowledge of the original version, translated the Greek version back into the original language. Then, a review panel, including experts in both languages and the subject matter, compared the back-translated version with the original to identify and resolve discrepancies. Further, to ensure content validity, we calculated the level of agreement between the translators and the expert panel [56]. We used the content validity index (CVI), which assesses each item for its relevance, clarity, and simplicity. An agreement rate above 80% was achieved for all items, confirming that the Greek version was conceptually equivalent to the original. Any items with discrepancies were discussed and revised to reach a consensus [57]. Following translation, we conducted a pilot study with a sample of ten participants per department to assess the reliability of the Greek version of the questionnaires. We evaluated internal consistency using Cronbach’s alpha, which demonstrated satisfactory reliability (α = 0.8), comparable to the original version [58].

#### 2.1.3. Statistical Analysis

In this study, missing data were not observed since all questions should be answered in order to submit the questionnaire and were one- or multiple-choice questions. Thus, no false or inaccurate responses were to be found within the dataset. The reported response rates reflect the final rates. A total of 271 students from the Departments of Dentistry and Nursing at the National and Kapodistrian University of Athens completed the survey, representing the final, usable dataset. Importantly, there were no missing data in the final responses, as all participants completed each section of the questionnaire in full. Data analysis was performed with the statistical package IBM SPSS v.28. Cronbach’s alpha indices were calculated to examine the reliability of the scales and subscales included in the questionnaire. Demographics of participating students were presented with absolute and relative frequencies (N%), while quantitative variables were summarized with descriptive statistics (M, SD). Distributions were examined in terms of normality via skewness and kurtosis and were considered normally distributed [59]. The association between categorical variables was examined with chi-square tests. The chi-square test was chosen because it is the most suitable method for examining relationships or differences between categorical demographic variables that we present below. This test allowed us to explore whether there were significant differences in the distribution of demographic characteristics across different groups in our study. Also, differences in psychological variables and resilience between departments and demographics were investigated with independent samples *t*-test and one-way analysis of variance (ANOVA). To determine the factors that had the most substantial effects on resilience, hierarchical multiple linear regression analyses were performed for the total samples as well as for the samples of each department, adjusting for demographics, after-graduation plans, and specialization [60].

## 3. Results

In the Department of Dentistry, 145 students out of 865 students responded (780 undergraduate and 85 postgraduate students) (response rate = 16.8%). In the Department of Nursing, 126 out of 1138 students responded (808 undergraduate and 330 postgraduate students) (response rate = 11.1%). Due to this result, we further conducted a comparison between the demographic characteristics of the respondents and those of the overall student population (age, gender, program, year of study, etc.), for which we have access to institutional demographic data. This allowed us to assess that there were no significant differences between the two groups.

Cronbach’s alpha indicators (Cronbach’s a) were calculated to examine the reliability of the scales and subscales included in the questionnaire. Our data provided the following: Cronbach’s alpha: Stress 0.879, Anxiety 0.854, Depression 0.876, Hope 0.759, Resilience (BRS 0.848, Resilience RAQ 0.762, Spiritual well-being 0.879, Stress somatization 0.750).

The total sample of this study consisted of 271 students: in the nursing (n = 126, 46.5%) and the dentistry (n = 145, 53.5%) departments (68.60% were female, 80.10% were undergraduate, 48.30% had Athens (the capital of Greece) as a place of origin, and 52.20% had a family income below EUR 25,000. Nursing students were mostly female, at a higher percentage (74.50%) compared to dentistry students (62.80%). Participating nurses were postgraduate students at 40.50%, versus 2.10% for the postgraduate students of the dentistry department. Most dentistry students originated from the capital city of Greece (52.40%) or other urban areas in Greece (15.20%), while nursing students reported the place of origin as the capital city of Greece at a lower rate (43.70%) as well as semi-urban or rural areas in mainland Greece (27.80%). Only 13.90% of nursing students reported a family income of over EUR 35,000, a significantly smaller percentage compared to 44.80% for dentistry students (Table 1).

Μost dentistry students (57.9%) will pursue postgraduate studies in their field after graduation or work as an employee in a dental office to gain experience (24.1%). Only 8.3% are considering opening their dental practice immediately after graduation, and only 3.5% reported that they will not practice dentistry by continuing their studies in other disciplines. As for nursing students, most of them are pursuing postgraduate studies (33.3%), followed by those who are considering working in a public hospital (26.9%) or at a private clinic (19.8%) immediately after graduation. Yet, a significant portion of nursing students (19.8%) reported that they will not practice nursing, either by continuing their studies in another discipline or following another profession.

General dentistry (20%), orthodontics (19.3%), prosthetics/implants (12.4%), and periodontology (10.3%) are the most common dental fields, followed by dentistry students. Nursing students have chosen mostly the specializations of emergency nursing (23%), surgical nursing (19.8%), psychiatric nursing (19.1%), and community nursing (12.7%).

Table 2 presents stress, anxiety, and depression levels and raw scores of DASS21 subscales for the total sample of students, as well as the differences between the samples of the dentistry and nursing departments. Overall, 48.70%, 51.30%, and 53.50% of participants presented some level of stress, anxiety, and depression symptoms, respectively. Specifically, severe or extremely severe symptoms were reported for stress by 21.3%, anxiety by 29.9%, and depression by 18.1% of participants. Stress levels were significantly associated with the department [χ^2^(4) = 9.69, *p* = 0.046], i.e., dentistry students were more likely to fall into the severe (16.6%) or extremely severe (9.7%) stress category, compared to nursing students (11.9% and 1.6%, respectively). Stress scores [t(267) = 2.18, *p* = 0.030, Cohen’s d = 0.26] and anxiety scores [t(267) = 2.03, *p* = 0.046, Cohen’s d = 0.24] were significantly different between departments, with higher mean scores of stress and anxiety for dental students compared to nursing students.

Table 3 presents resilience, hope, and spiritual well-being scores for the total sample and the samples of the two departments. Overall, the sample presented scores of resilience at the lower end of the normal range (M = 3.42, SD = 0.79), hope scores, and spiritual well-being scores (M = 2.38, SD = 0.76) at a moderate level with no significant differences between departments. There was a weak but significant difference between departments in the determination factor of resilience [t(269) = 2.13, *p* = 0.034, Cohen’s d = 0.26], with dentistry students being slightly more determined than nursing students. Moreover, organization [t(269) = 2.22, *p* = 0.027, Cohen’s d = 0.27] and relationship factors [t(269) = −2.89, *p* = 0.04, Cohen’s d = 0.35] were also higher for dentistry students. On the other hand, the problem-solving factor was higher in nursing students [t(269) = −2.03, *p* = 0.043, Cohen’s d = 0.25].

Τhe results of *t*-tests for the differences between male and female participants per educational level in psychological variables showed that male postgraduate students reported significantly higher resilience compared to females [t(52) = 2.39, *p =* 0.020, Cohen’s d = 0.97] while female undergraduate students showed higher scores of stress [t(215) = 3.96, *p <* 0.001, Cohen’s d = 0.56], and anxiety [t(193) = −4.30, *p <* 0.001, Cohen’s d = 0.57], and lower levels of resilience [t(215) = −3.43, *p <* 0.001, Cohen’s d = 0.49], compared to male undergraduate students.

Further, family income was significant for stress, with lower values of stress for the EUR 25.001–35.000 income group and higher values of stress for both lower income (<EUR 25.000) and higher income levels (>EUR 35.000). Yet, significant at *p <* 0.05 was only the difference between the EUR 25.001–35.000 and EUR 35.001–50.000 family income groups.

Finally, no significant differences between place of origin groups were detected, meaning that stress, anxiety, depression, and resilience do not vary according to place of origin.

Table 4 presents the results of hierarchical multiple linear regression for the factors that affect resilience in the total sample. Resilience is the dependent variable, as this analysis investigates how various factors (hope, stress, anxiety, etc.) predict or influence resilience levels. So, hope was a significant predictor of higher resilience (β = 0.394, *p <* 0.001), while stress (β = −0.174, *p <* 0.05) and anxiety (β = −0.152, *p <* 0.05) were associated with lower resilience levels after adjusting for demographics (department, educational level, and gender). Yet, hope was the only significant predictor of the resilience of dentistry (β = 0.434, *p <* 0.001) and nursing students (β = 0.426, *p <* 0.001) when the samples were examined separately.

## 4. Discussion

This study aimed to investigate anxiety, stress, resilience, hope, and spiritual well-being among dentistry and nursing students. The sample consisted of students from the Department of Dentistry and Nursing at the National and Kapodistrian University of Athens. Most participants were female and undergraduate students, with a significant portion from the capital, Athens. Nursing students were predominantly female compared to dentistry students, and there was a higher proportion of postgraduate students among nurses. Overall, more than half of the participants experienced anxiety, with dentistry students showing higher levels than nursing students. Factors such as gender, educational level, and family income were significantly linked to anxiety levels.

### 4.1. Overall Levels of Stress, Anxiety, and Depression Among Dentistry and Nursing Students

The present survey indicated that half of nursing and dentistry students experienced at least mild levels of stress (for the overall sample: 48.7%, for dentistry: 51%, and for nursing: 46%), anxiety (for the overall sample: 51.3%, for dentistry: 53.8%, and for nursing: 48.4%), and depression (for the overall sample: 53.5%, for dentistry: 55.2%, and for nursing: 51.6%). The prevalence of depression was the highest in our sample, followed by anxiety and stress. However, it is particularly significant that one-third of students experienced severe and very severe levels of stress (for the overall sample: 29.9%, for dentistry: 34.5%, and for nursing: 24.6%), while 2 in 10 students experienced severe and very severe stress (for the overall sample: 20.3%, for dentistry: 26.3%, and for nursing: 13.5%) and depression (for the total sample: 18%, for dentistry: 20%, and for nursing: 15.9%). These rates are higher than in other studies on Greek nursing students during the first wave of the COVID-19 pandemic [4] and frontline nurses in referral hospitals for COVID-19 [61]. It is also noteworthy to observe that working nurses experience anxiety while nursing students experience depression at a higher rate. According to Kapourani et al. (2020) [62], the prevalence of clinical depression among university students is 2.5–3 times higher than the 5–6% expected prevalence in the general population. Furthermore, compared to the 1.23% estimated among the general population [63], there was an approximately eight-fold increase in suicidal ideation, with 9.7% currently thinking about suicide and making precise plans on how to accomplish it. Suicide ideation was found in 16.7% of medical Greek students, while the use of sleeping pills in the previous month was reported by 8.8% [64]. Furthermore, according to Eleftheriou et al. (2021), 8.8% of Greek medical students reported using sleeping drugs in the previous month, while 16.7% reported having suicidal thoughts [64].

As for dentistry students, in contrast to the Harris et al. (2018) study [65], which showed that dentistry students’ anxiety, stress, and depression fell between a normal and a moderate range, and the study of Zenthöfer et al. (2023) [66], where stress and anxiety levels among dentistry students were moderate both during the university breaks and during the academic year, in our study dentistry students experienced high-level stress. This finding was also reported in the Deeb et al. (2018) survey [67], which showed that 9% of dentistry students had depression levels above the moderate range, and the Ahad et al. (2021) study [15], where the prevalence of anxiety in dentistry students was also highest, followed by depression and stress. The present study also found that dentistry students experience higher stress, anxiety, and depression levels compared to nursing students, as reported elsewhere [18].

Overall, those majoring in dentistry, nursing, and other health sciences reported higher levels of tension and anxiety than those not pursuing health-related careers, as already mentioned elsewhere [68,69]. For example, among European college students, the prevalence of depression, anxiety, and stress was 39%, 47%, and 35.8%, respectively [70], while our data reveal higher percentages in all three parameters for both dentistry and nursing students. Like dentistry students’ high levels of stress [71], nursing students have also been shown to experience high levels of anxiety and depression [41,72], as well as high levels of stress [40,73,74], as also confirmed in our study.

### 4.2. Factors Affecting Stress, Anxiety, and Depression of Both Dentistry and Nursing Students

#### 4.2.1. Gender

Female undergraduate students showed higher scores of stress and anxiety compared to male undergraduate students. According to Dafogianni et al. (2022) [4], female students experienced noticeably higher levels of stress and anxiety than males. Patsali et al. (2020) [75] suggested that female students were at double the risk of developing depression in comparison to males. In the study of Eleftheriou et al. (2021) [64] on Greek medical students, a comparison between genders revealed that females had noticeably more severe symptoms across the board for all mental health metrics. Results from previous research on university students [76,77] and in the general population [78,79] support that conclusion.

The available research on the relationship between gender and mental health is not entirely conclusive. In Xie et al. (2021) [80] study, males reported depressed symptoms more frequently, and Liu et al. (2020) [81] observed no statistically significant difference in anxiety or depression between the genders. However, according to Batra et al. (2021) [82], in a systematic review, female students had higher stress and anxiety levels. Our study’s findings are supported by these outcomes. Thus, various elements related to the environment, genetic background, and physiological makeup have been proposed as influences on the variations in mental health issues between genders [83,84].

#### 4.2.2. Department and Educational Level (Undergraduate, Postgraduate)

Stress levels were significantly associated with the department; i.e., dentistry students were more likely to fall into the severe (16.6%) or extremely severe (9.7%) stress category, compared to nursing students (11.9% and 1.6%, respectively). Stress scores and anxiety scores were significantly different between departments, with higher mean scores of stress and anxiety for dentistry students compared to nursing students. There was no significant difference between educational level and the levels of anxiety, stress, and depression. Simegn et al. (2021) [76] concluded that students from non-health-related departments were significantly associated with anxiety, while students attending 1st and 2nd years were significantly associated with stress. The same findings were reported by Labrague et al. (2018) [85], who discovered that nursing students’ years of study had a significant impact on their total stress experiences. Senior students perceived less stress than junior students did. Years of study and social support were determinants of anxiety [77], while academic performance [86] and age (Shah et al., 2021) [87] were associated with stress. The reason why the percentage of mental health issues reported by nursing students was likely lower than that of dentistry students in the current study is likely because the nurses who participated were postgraduate students, making up 40.50% of the participants, compared to 2.10% of the dentistry department postgraduate students.

Furthermore, university students are more likely than the general population to experience mental health issues, and numerous studies have shown that this is the case [88,89,90]. The prevalence of mental health problems among college students may be caused by a wide range of intricately linked causes. They deal with a variety of stressors, including changes in life stages, worries about the future, accommodation issues, academic pressure from exams, study loads, and intense pressure to perform well at university [91,92].

Furthermore, the demanding requirements of clinical training and the development of complex communication skills to deal with patients are important sources of increasing stress among dental students [11,71,93,94,95,96,97], while Aloufi et al. (2021) [39] suggest that stress, anxiety, and depression should be decreased in nursing students. However, it has been noted that nursing students have higher stress levels and more associated physical and psychological symptoms than students in other health-related fields [98]. A range of issues, such as first-hand hospital experience, clinical assignments and coursework, nursing skills practices, evaluation and clinical examinations, and connections with patients, families, and other health professionals, can result in clinical stressors for nursing students. Furthermore, other stressors include gaps in theory and practice, fear of making a mistake and unknown events, dealing with equipment, and interacting with patients, peers’ staff, and faculty members [99,100,101]. Stress can cause illness, health problems, poor academic performance, and program withdrawal among nursing students. These outcomes can then have an impact on the standard of patient care [102,103].

On the impact of the educational level on the issue discussed here, we can mention the study of Antoniadou et al. (2023) [46] in academic dentistry and nursing personnel that showed that education had a significant moderate–strong effect on psychological health, while the department had a significant moderate effect on social ties. Participants in both departments who were PhD-level showed better psychological health than those who were less educated. Results showed that PhD individuals’ psychological health was generally better. Also, numerous studies looking at stress in dentistry undergraduate students have found that stress levels significantly rise with each year of education [104]. Alzahem et al. (2011) [105] divided stress into five categories in their systematic review: living arrangements, educational setting, personal, academic, and clinical issues. One of the most stressful aspects, according to several reports, is not having enough time for leisure and studying for exams [11,105], as mentioned by our participants. Furthermore, studies have shown that stresses are influenced by geographical and cultural characteristics and are not universally equal [105,106]. Gender, academic year, marital status, the first choice of admission, money issues, housing situation, exams and grades, workload, and patients are the stressors that are most frequently mentioned [106,107,108,109,110], as presented here.

#### 4.2.3. Family Income

Family income was significant for stress in our study, with lower values of stress for the EUR 25.001–35.000 income group and higher values of stress for both lower income (<EUR 25.000) and higher income levels (>EUR 35.000). Adjepong et al. (2022) [111] found that financial stress was positively correlated with repetitive negative thinking, negative mood, and anxiety. In the systematic review by McCloud et al. (2019) [112], several financial stress indicators had varying outcomes. For instance, financial challenges such as bill payment difficulties or stress related to debt concerns were more reliable indicators of mental health disorders than debt quantity. The study by Da Silva et al. (2022) [71] demonstrated that dentistry students with low family incomes presented higher levels of anxiety and stress, as was also the case in our study. Furthermore, students’ well-being may be significantly impacted by the financial strain of their education [113]. Nonetheless, more research is required to understand how students’ financial circumstances affect their academic and health performance.

### 4.3. Resilience Level of Both Dentistry and Nursing Students

As regards resilience, the present study indicated that it appeared to be at the lower end of the normal range, whereas hope and spiritual well-being scores appeared to be at a moderate level, with no significant differences between departments, in comparison to a recent study (Schwitters and Kiesewetter, 2023) [20], which demonstrated that dentistry students have moderate levels of resilience and lower levels of resilience than medical students. In another study (Smith et al., 2020) [21], dentistry students demonstrated relatively high levels of resilience, and similarly, the study by Harris et al. (2018) [65] showed high levels of well-being among dentistry students. The study by Schwitters and Kiesewetter (2023) [20] displayed these effects lie in emotional exhaustion and decreased personal performance. In the current study, the determination factor of resilience, organization, and relationships’ factors was higher for dentistry students, and the problem-solving factor was higher for nursing students.

Moderate to high degrees of resilience were found in the Labrague (2021) [85] study, which lessened the detrimental effects of COVID-associated stress on psychological well-being and life satisfaction. In a similar vein, Dafogianni et al. (2022) [4] found that stress, anxiety, and depression were significantly negatively connected with resilience, life orientation, and active/positive coping and positively correlated with negative affect scores. In our study, stress and anxiety were associated with lower resilience levels. Pineda et al. (2022) [114] investigated the association between social support, personal resilience, and quality of life in nursing students and discovered that most of them had normal levels of resilience, which improved the participants’ quality of life. These results support our data and prove the usefulness of resilience as a stress-reduction strategy, emphasizing the need to help university students develop resilience to minimize the damaging effects of stressors.

Furthermore, the study by Montas et al. (2021) [19] indicated resilience influences academic achievement positively, while the results of another study (Maragha et al., 2023) [12], which concerned dentistry students, found that ethnic origins and major life events, such as the COVID-19 pandemic, influenced resilience levels. Following the review by Smith et al. (2023) [35], nursing students from all around the world have experienced high levels of stress during the COVID-19 pandemic. Almost unanimously, resilience was identified as a key protective factor against stress and the development of psychological morbidity. Nursing students with higher levels of resilience were deemed more likely to continue their studies, despite COVID-related challenges. The development of resilience levels has the potential to empower nursing students for academic and clinical success. Therefore, it is important to integrate resilience development activities into undergraduate nursing curricula [35].

#### 4.3.1. Factors Affecting Resilience in Dentistry and Nursing Students

In the present study, male postgraduate students reported significantly higher resilience compared to females, whilst female undergraduate students displayed higher scores of stress and anxiety compared to male undergraduate students, such as the study by Dafogianni et al. (2022) [4], which demonstrated high anxiety and stress levels of Greek nursing students during the COVID-19 pandemic, with increased levels of anxiety, stress, and negative affect score in females and increased levels of resilience in males, and as the study by Da Silva et al. (2022) [71], with greater anxiety scores in females compared to males. Moreover, the study by Da Silva et al. (2022) [71] pointed out that a higher academic semester was associated with higher anxiety and stress levels, particularly in the final academic year. When compared to female students, male undergraduate and postgraduate students showed much greater resilience. In a study by Smith et al. (2020) [21], resilience was significantly associated with gender, with females showing more resilience than males and overall health. Adjepong et al.’s study (2022) [111], in which female students reported higher stress levels and demonstrated lesser resilience and higher negative mood ratings, came to the same conclusion.

In contrast to the study by Maragha et al. (2023) [12], which showed that dentistry students influenced resilience levels related to ethnic origins, no significant differences between place of origin groups were detected in the present study. Hence, depression, anxiety, stress, and resilience do not vary according to place of origin, according to our findings.

Moreover, in our study, stress and anxiety were linked to lower levels of resilience, whereas hope was a strong predictor of better resilience. However, the only significant predictor of resilience among nursing and dentistry students was hope. Fewer symptoms of stress, anxiety, and sadness were substantially correlated with more resilience and optimism [4]. The study by Da Silva et al. (2022) [71] among dentistry students presented that higher social support was associated with lower anxiety, and lower anxiety was linked with a better quality of life. Therefore, this study suggested a healthier academic environment and student educational programs for reducing stress and anxiety levels [71]. Park et al. (2022) [115] found that problem-solving was the most popular coping method among university students, followed by seeking out social support and avoidance.

Psychopathological symptomatology was further predicted by the presence of stressors and an avoidant coping style, but a greater level of life satisfaction was predicted by an active coping style that controlled such stressors [116]. Ziarko et al. (2020) [117] demonstrated resilience’s mediation role in the process of choosing coping strategies based on the demands of a particularly challenging circumstance. Although young adults extensively utilized avoidance throughout the pandemic [118], their coping mechanisms may change depending on the sociocultural situation [119].

In the Dafogianni et al. (2022) study [4], older students, those engaging in behavioral withdrawal and avoidance coping, and individuals with higher negative affect scores, lower positive affect scores, and lower optimism showed significantly higher indicators of depression. Additionally, students who frequently used drugs relied on negative emotions as coping mechanisms, had higher negative affect scores, were less resilient, and exhibited more worry and tension, as also confirmed by our findings. Fourth-year students employed more proactive and constructive coping mechanisms than first- or second-year students, as was also the case in our study. Previous research identified age and academic year as key predictors of stress and coping strategies [61,85,98]. Also, in a study of Greek dentistry and nursing academic personnel [46], dentistry department participants showed higher social interaction QOL across all educational categories compared to nursing department individuals. Possibly, these results suggest a correlation between academic staff and students, indicating that staff resilience and quality of life might influence student resilience and mental health, too. Therefore, interventions to enhance staff mental resilience and quality of life could indirectly benefit students. Further research could explore whether certain personality profiles influence the choice of nursing and dentistry careers for students.

#### 4.3.2. Targeted Interventions and Improved Communication Strategies in Dentistry and Nursing Education

This study emphasizes the urgent need for targeted interventions and enhanced support systems to promote the well-being of dental and nursing students. Key areas identified by students include strengthening psychological services, improving communication with teaching staff, extending internship durations to increase hands-on experience, and revising certain courses to better meet the demands of their fields [120,121]. Effective communication between students and educators also plays a pivotal role in reducing stress and enhancing academic support [122]. In healthcare education, enhancing open communication skills can significantly improve student experiences. An interprofessional model that encourages open communication among healthcare professionals could be a valuable framework for managing stress and increasing job satisfaction among students in both disciplines [123]. Additionally, the study underlines the need for more flexible academic procedures and stronger support systems to address students’ unique challenges. Implementing resilience-building strategies, such as stress management techniques and emotional regulation training, is essential for improving student well-being. Increasing acceptance of coaching has also been linked to better stress management outcomes and overall student success [124,125,126,127,128]. Antoniadou et al. (2024) [129] emphasize further the effectiveness of coaching in managing stress and controlling somatization symptoms among students in high-pressure healthcare education settings. It seems that by offering tailored guidance and enhancing personal growth, coaching equips students with essential skills for stress management and emotional regulation in both dentistry and nursing educational settings. This personalized support not only enhances their well-being but also improves their academic performance, strengthening the importance of integrating coaching into comprehensive support systems for student success in these two disciplines. In addition to coaching, the role of faculty support in enhancing psychological resilience is critical for helping students face academic challenges effectively. Labrague et al. (2024) [130] also identify psychological resilience as a key mediator between supportive nurse faculty and the clinical adjustment of student nurses. This finding highlights the importance of building resilience through institutional support mechanisms, including mentorship and stress management programs, to ensure students adapt successfully to demanding environments. Integrating such strategies into nursing education not only enhances students’ capacity to cope with stress but also strengthens their ability to thrive in clinical settings, aligning with broader efforts to promote well-being and academic success in healthcare environments.

### 4.4. Limitations of the Present Study

The present study has certain limitations. The sample was composed exclusively of dentistry and nursing students from the Dentistry and Nursing Departments at the University of Athens. As a result, the findings should not be generalized to undergraduates and postgraduates in other non-health-related subjects. Additionally, the sample was predominantly female, and most nursing students were postgraduates, whereas most dentistry students were undergraduates. These factors may limit the generalizability of the results. Despite these limitations, the results provide a valuable overview of the current resilience levels and coping mechanisms among dentistry and nursing students. This study highlights specific areas where targeted interventions can be developed to support these students better. Moreover, the findings highlight the importance of addressing emotional exhaustion and personal performance issues within these student populations, offering a foundation for future research and program development aimed at enhancing student well-being and academic success. The cross-sectional research methodology is another limitation, as it captures responses at a single point in time. Given the dynamic nature of the variables under investigation, longitudinal research would be beneficial in monitoring changes over time. Furthermore, this study had a low response rate, which could impact the findings as the experiences of participants may differ from those of those who did not participate. To overcome this limitation, future research will enhance recruitment efforts by using multiple channels, offering incentives to motivate participation, and sending follow-up reminders. Additionally, a longitudinal study design should be implemented to track changes over time, and qualitative methods such as semi-structured interviews or focus groups will be incorporated to provide deeper insights and complement the quantitative data, which is in our future research plans. This approach will provide a more comprehensive understanding of the factors that positively and negatively influence the development of resilience in this population. Additionally, future studies could include a more diverse and balanced sample, encompassing various academic disciplines and a more equal gender distribution, to enhance the generalizability of the findings.

## 5. Conclusions

The present study indicates that anxiety is prevalent among dentistry and nursing students, affecting more than half of the sample, with dentistry students reporting higher anxiety than nursing students. Anxiety was further substantially correlated with factors like family income, gender, and educational attainment, which influence its manifestation. Resilience was positively predicted by hope and negatively associated with stress and anxiety. Promoting students’ well-being and academic success requires effective stress-reduction and resilience-building techniques aimed to not only improve student performance but also support future healthcare professionals’ personal sustainability and holistic growth.

## Figures and Tables

**Table 1:** Demographic characteristics of the sample of students in total and per Department of Dentistry and Nursing and associations between department and demographics.

		Total (N = 271)		Department			
				Dentistry (n = 145, 53.5%)		Nursing (n = 126, 46.5%)	
		N	%	N	%	N	%
Gender	Male	85	31.40%	54	37.30%	31	24.60%
	Female	186	68.60%	91	62.80%	95	75.40%
Educational level	Postgraduate studies/PhD	54	19.90%	3	2.10%	51	40.50%
	Undergraduate studies	217	80.10%	142	97.90%	75	59.50%
Place of origin	Athens (Capital of Greece)	131	48.30%	76	52.40%	55	43.70%
	Another country, not Greece	19	7.00%	15	10.30%	4	3.20%
	Another city in Greece	40	14.80%	22	15.20%	18	14.30%
	Mainland Greece (towns and villages)	48	17.70%	13	9.00%	35	27.80%
	Greece Island Region (towns and villages)	33	12.20%	19	13.10%	14	11.10%
Family income	<EUR 15,000	32	16.20%	14	14.60%	18	17.80%
	EUR 15,001–25,000	71	36.00%	24	25.00%	47	46.50%
	EUR 25,001–35,000	37	18.80%	15	15.60%	22	21.80%
	EUR 35,001–50,000	34	17.30%	24	25.00%	10	9.90%
	>EUR 50,000	23	11.70%	19	19.80%	4	4.00%

**Table 2:** Stress, anxiety, and depression levels and raw scores of DASS-21 for the total sample and differences between the samples of the two departments.

		Total (N = 271)		Department			
				Dentistry (n = 145, 53.5%)		Nursing (n = 126, 46.5%)	
		Ν	%	Ν	%	Ν	%
Stress levels ^1^	Normal	139	51.3%	71	49.0%	68	54.0%
	Mild	32	11.8%	14	9.7%	18	14.3%
	Moderate	45	16.6%	22	15.2%	23	18.3%
	Severe	39	14.4%	24	16.6%	15	11.9%
	extremely severe	16	5.9%	14	9.7%	2	1.6%
Anxiety levels ^2^	Normal	132	48.7%	67	46.2%	65	51.6%
	Mild	28	10.3%	12	8.3%	16	12.7%
	Moderate	30	11.1%	16	11.0%	14	11.1%
	Severe	31	11.4%	18	12.4%	13	10.3%
	extremely severe	50	18.5%	32	22.1%	18	14.3%
Depression levels ^3^	Normal	126	46.5%	65	44.8%	61	48.4%
	Mild	30	11.1%	12	8.3%	18	14.3%
	Moderate	66	24.4%	39	26.9%	27	21.4%
	Severe	27	10.0%	12	8.3%	15	11.9%
	extremely severe	22	8.1%	17	11.7%	5	4.0%
Raw scores of DASS-21 subscales (M, SD)	Stress ^4^	7.96	5.10	8.58_a_	5.58	7.25_b_	4.41
	Anxiety ^5^	5.14	4.67	5.66_a_	5.10	4.55_b_	4.05
	Depression	5.98	4.86	6.40_a_	5.33	5.49_a_	4.23
	Stress somatization	9.56	5.62	9.96_a_	5.93	9.10_a_	5.22

Note: 1. Significant difference between stress levels and department (χ^2^(4) = 10.38, *p* = 0.034); 2. Non-significant difference between anxiety level and department (χ^2^(4) = 4.15, *p* = 0.386); 3. Non-significant difference between depression level and department (χ^2^(4) = 9.10, *p* = 0.059); 4. Significant difference between departments in stress scores (t(267) = 2.18, *p* = 0.030, Cohen’s d = 0.26); 5. Significant difference between departments in anxiety scores (t(267) = 2.03, *p* = 0.046, Cohen’s d = 0.24); Values in the same row and subtable not sharing the same subscript are significantly different at *p* < 0.05 in the two-sided independent samples *t*-tests.

**Table 3:** Resilience, hope, and spiritual well-being for the total sample and differences between the samples of the two departments.

	Total (N = 271)		Department			
			Dentistry (n = 145, 53.5%)		Nursing (n = 126, 46.5%)	
	M	SD	M	SD	M	SD
Brief Resilience Scale (BRS) score	3.42	0.79	3.45_a_	0.82	3.39_a_	0.76
Resilience assessment questionnaire						
- Self-awareness	3.14	0.86	3.16_a_	0.91	3.12_a_	0.80
- Determination ^1^	3.97	0.85	4.07_a_	0.86	3.85_b_	0.83
- Vision	2.93	0.97	2.99_a_	1.00	2.87_a_	0.92
- Self-confidence	2.77	1.05	2.83_a_	1.08	2.71_a_	1.03
- Organization ^2^	3.35	1.22	3.50_a_	1.24	3.17_b_	1.18
- Problem-solving ^3^	3.30	1.20	3.17_a_	1.17	3.46_b_	1.22
- Interaction	3.69	1.05	3.72_a_	1.07	3.66_a_	1.04
- Relationships ^4^	4.34	0.80	4.47_a_	0.74	4.19_b_	0.85
- Hope	5.23	1.03	5.24_a_	1.07	5.21_a_	0.98
- Spiritual well-being	2.38	0.76	2.40_a_	0.76	2.36_a_	0.77

Note: 1. Significant difference between departments in determination (t(269) = 2.13, *p* = 0.034, Cohen’s d = 0.26); 2. Significant difference between departments in organization (t(269) = 2.22, *p* = 0.027, Cohen’s d = 0.27); 3. Significant difference between departments in problem-solving (t(269) = −2.03, *p* = 0.043, Cohen’s d = 0.25); 4. Significant difference between departments in relationships (t(269) = −2.89, *p* = 0.04, Cohen’s d = 0.35); Values in the same row and subtable not sharing the same subscript are significantly different at *p <* 0.05 in the two-sided independent samples *t*-tests.

**Table 4:** Hierarchical multiple linear regression results for the factors that affect resilience in the total sample and the department samples.

		**Total Sample**				**Dentistry Students**				**Nursing Students**			
**Model**	**Independent Variables**	**B**	**SE**	**β**	***p***	**B**	**SE**	**Β**	***p***	**B**	**SE**	**β**	***p***
1	(Constant)	2.166	0.263		<0.001	2.033	0.361		<0.001	2.496	0.393		<0.001
	Stress	−0.027	0.012	−0.177	0.022	−0.028	0.016	−0.189	0.084	−0.036	0.017	−0.213	0.042
	Anxiety	−0.027	0.012	−0.157	0.024	−0.026	0.015	−0.160	0.094	−0.029	0.018	−0.161	0.100
	Depression	−0.011	0.012	−0.069	0.361	−0.007	0.016	−0.044	0.675	−0.017	0.018	−0.099	0.347
	Hope	0.315	0.046	0.409	<0.001	0.321	0.065	0.416	<0.001	0.319	0.067	0.417	<0.001
	Spiritual well-being	0.014	0.062	0.014	0.818	0.068	0.088	0.062	0.441	−0.105	0.089	−0.109	0.240
2	(Constant)	2.255	0.276		<0.001	1.631	0.399		<0.001	2.403	0.455		<0.001
	Stress	−0.027	0.012	−0.174	0.026	−0.026	0.016	−0.174	0.113	−0.029	0.018	−0.170	0.117
	Anxiety	−0.026	0.012	−0.152	0.030	−0.027	0.016	−0.169	0.089	−0.023	0.019	−0.124	0.230
	Depression	−0.014	0.012	−0.085	0.264	−0.005	0.017	−0.035	0.742	−0.024	0.019	−0.137	0.211
	Hope	0.303	0.047	0.394	<0.001	0.334	0.067	0.434	<0.001	0.326	0.071	0.426	<0.001
	Spiritual well-being	0.015	0.062	0.014	0.812	0.086	0.092	0.079	0.353	−0.113	0.094	−0.118	0.229
	Department: Dentistry vs. Nursing	0.101	0.083	0.064	0.223	-				-			
	Education level: Post- vs. Undergraduate	−0.026	0.107	−0.013	0.808	0.237	0.341	0.041	0.489	0.326	0.071	0.426	0.983
	Gender: Female vs. Male	−0.096	0.083	−0.056	0.246	−0.014	0.107	−0.008	0.897	0.003	0.139	0.002	0.058
	Dentists’ specialization												
	General dentistry					0.013	0.141	0.006	0.925				
	Orthodontics					0.158	0.134	0.076	0.241				
	Periodontology					0.358	0.174	0.133	0.041				
	Prosthetic implants					0.393	0.155	0.158	0.012				
	After graduation plans												
	Practice dentistry as an employee in a dental office					0.215	0.153	0.112	0.221				
	Pursue postgraduate studies in dentistry					0.178	0.14	0.180	0.140				
	Nurses’ specialization												
	Emergency nursing									0.195	0.169	0.113	0.252
	Community nursing									0.437	0.205	0.202	0.035
	Surgical nursing									0.131	0.173	0.068	0.452
	Psychiatric nursing									0.269	0.184	0.146	0.145
	After graduation plans												
	Pursue postgraduate studies in nursing									0.078	0.137	0.049	0.572
	Will not practice nursing									0.058	0.155	0.035	0.712

## Data Availability

The original contributions presented in the study are included in the article; further inquiries can be directed to the corresponding authors.

## Appendix A. Information About Components of the Questionnaire and Study Tools

DASS-21

Description of the Tool

For this study, we will use the DASS-21 tool (Lovibond and Lovibond, 1995) [47], a shorter version of the full survey (DASS-42). It consists of three self-report scales designed to measure the negative emotional states of depression, anxiety, and stress. Each of them contains seven items. Participants respond using a 4-point severity and frequency scale to rate the extent to which they had experienced each in the past week: 0 = did not apply to me at all, 1 = applied to me to some extent, or some of the time, 2 = applied to me to a significant extent, or much of the time, and 3 = applied to me very much, or most of the time. Separate scores for depression, anxiety, and stress are calculated by summing the scores for each. Then, they are multiplied by two to match the DASS 42 scale. The DASS 21 scale has been translated and validated in the Greek population [52,131].

FACIT-Sp-12

Description of the tool

The most widely validated questionnaire for measuring spirituality is the functional assessment of chronic illness therapy–spiritual well-being 12 (FACIT-Sp-12) scale–non-illness [48]. The scale was validated and measured for reliability and validity in Greek [53]. The internal consistency of the scale was rated highly satisfactory (Cronbach’s a = 0.789), and statistical analysis revealed three factors: faith, meaning, and peace. The tool is demonstrated in Appendix B. The FACIT-Score can be found at the following link: https://www.facit.org/measures/facit-sp-12 (accessed on 30 March 2024).

ADULT HOPE SCALE (AHS) (Snyder, 1991).

Description of the tool

The adult hope scale is a 12-item tool that identifies a respondent’s level of hope. Specifically, the scale is divided into two subscales that comprise Snyder’s cognitive model of hope: (1) self-action (i.e., goal-directed action) and (2) pathways (i.e., planning to achieve goals). Of the 12 elements, 4 compose the agency subscale, and 4 constitute the Pathways subscale. The remaining four elements are complementary. Each item is answered using an 8-point Likert-type scale ranging from definitely false to definitely true. It should be noted that the authors recommend that when administering the scale, it should be called “The Future Scale” (https://ppc.sas.upenn.edu/resources/questionnaires-researchers/adult-hope-scale) (accessed on 30 March 2024).

Researchers can either examine the results at the subscale level or combine the two subscales to create an overall hope score. There is no specific score that defines high and low hope. However, an early study by the author of the AHS suggested that “high hope” and “low hope” equated to a combined agency and pathway score of >60 and <35, respectively [87,132,133,134].

Resilience Assessment Questionnaire (RAQ8)

The resilience assessment questionnaire was created by psychologist Derek Mowbray (2011) [50]. In our study, the short form of the questionnaire is used (8 questions) (RAQ8). The longer version consists of 40 questions (RAQ 40). The RAQ8 has been examined for reliability and validity [50].

Brief Resilience Scale (BRS)

The Brief Resilience Scale (BRS) [51] was among the scales with the most satisfactory psychometric properties. Moreover, it was evaluated [135] as one of the most frequently used resilience scales in a total of 25 scales [54]. Kyriazos et al. (2018) [54] examined the reliability and validity of the Brief Resilience Scale in the Greek population, with a satisfied Cronbach’s indicator (a = 0.80–0.91).

## Appendix B. Questionnaire

PART ONE

Q1.What is your gender?

Q2. What is your level of education (1–5 years, degree/diploma) undergraduate, postgraduate, PhD, postdoctoral)

Q3. What is your place of origin?
  (1) Athens, (2) another urban center (capital of a prefecture), (3) continental region (towns and villages), (4) island region (towns and villages), (5) another country outside Greece

Q4. Which dental specialty would you like to pursue in the future? (for dental students)
  (1) General dentistry, (2) Pedodontics, (3) Orthodontics, (4) Oral surgery, (5) Dentistry-sensitive dentistry, (6) Endodontics, (7) Periodontology, (8) Stomatology/hospital dentistry, (9) Prosthetic implants, (10) I will not practice dentistry, (11) I am not a dental student

Q5. Which nursing specialization would you like to pursue? (for nursing students)
  (1) Community nursing, (2) Psychiatric nursing, (3) Surgical nursing, (4) Pathological nursing, (5) Emergency nursing, (6) Oncology nursing, (7) I will not practice nursing, (8) I am not a nursing student

Q6. What is your family’s annual income?
  P1. less than EUR 15.000, P2. EUR 15.001–25.000, P3. EUR 25.001–35.000, P4. EUR 35.001–50.000, P5. more than EUR 50.000, P6. I do not know, P7. I have never worried about this issue

Q7.1. What are you thinking of doing after graduation? (for dentistry students)
  P1. I will open my own clinic, P2. I will work as a clerk in a colleague’s office for a few years until I see what I can do, P3. I will work as a dentist abroad, P4. I will do postgraduate studies and work in a colleague’s office at the same time, P5. I will not practice clinical dentistry but will do something else in the field, P6. I will do something else professionally, P7. I will continue my studies in another discipline, P8. I am not a student of dentistry

Q7.2 What do you plan to do when you graduate? (for nursing students)
  P1. I will work in a private clinic, P2. I will work in a public hospital, P3. I will do postgraduate studies, P4. I will do something else professionally, P5. I will continue my studies in another science, P6. I am not a nursing student

PART TWO

DASS-21

Please read each statement and circle number 0, 1, 2, or 3, which indicates how much the statement applied to you over the past week. There are no right or wrong answers. Do not spend too much time on any statement. The rating scale is as follows: 0 = Did not apply to me at all; 1 = Applied to me to some degree, or some of the time; 2 = Applied to me to a considerable degree or a good part of the time; 3 = Applied to me very much or most of the time

1 (s)	I found it hard to wind down	0	1	2	3
2 (a)	I was aware of the dryness of my mouth	0	1	2	3
3 (d)	I could not seem to experience any positive feelings at all	0	1	2	3
4 (a)	I experienced breathing difficulty (e.g., excessively rapid breathing, breathlessness in the absence of physical exertion)	0	1	2	3
5 (d)	I found it difficult to work up the initiative to do things	0	1	2	3
6 (s)	I tended to over-react to situations	0	1	2	3
7 (a)	I experienced trembling (e.g., in my hands)	0	1	2	3
8 (s)	I felt that I was using a lot of nervous energy	0	1	2	3
9 (a)	I was worried about situations in which I might panic and make a fool of myself	0	1	2	3
10 (d)	I felt that I had nothing to look forward to	0	1	2	3
11 (s)	I found myself getting agitated	0	1	2	3
12 (s)	I found it difficult to relax	0	1	2	3
13 (d)	I felt downhearted and blue	0	1	2	3
14 (s)	I was intolerant of anything that kept me from getting on with what I was doing	0	1	2	3
15 (a)	I felt I was close to panic	0	1	2	3
16 (d)	I was unable to become enthusiastic about anything	0	1	2	3
17 (d)	I felt I was not worth much as a person	0	1	2	3
18 (s)	I felt that I was rather touchy	0	1	2	3
19 (a)	I was aware of the action of my heart in the absence of physical exertion (e.g., sense of heart rate increase, heart missing a beat)	0	1	2	3
20 (a)	I felt scared without any good reason	0	1	2	3
21 (d)	I felt that life was meaningless	0	1	2	3

FACIT-Sp-12 (Version 4)

Below is a list of statements that other people with your illness have said are important. Please circle or mark one number per line to indicate your response as it applies to the past 7 days.

		**Not at all**	**A little bit**	**Some-what**	**Quite** **a bit**	**Very much**
Sp1	I feel peaceful	0	1	2	3	4
Sp2	I have a reason for living	0	1	2	3	4
Sp3	My life has been productive	0	1	2	3	4
Sp4	I have trouble feeling peace of mind	0	1	2	3	4
Sp5	I feel a sense of purpose in my life	0	1	2	3	4
Sp6	I can reach down deep into myself for comfort	0	1	2	3	4
Sp7	I feel a sense of harmony within myself	0	1	2	3	4
Sp8	My life lacks meaning and purpose	0	1	2	3	4
Sp9	I find comfort in my faith or spiritual beliefs	0	1	2	3	4
Sp10	I find strength in my faith or spiritual beliefs	0	1	2	3	4
Sp11	My illness has strengthened my faith and spiritual beliefs	0	1	2	3	4
Sp12	I know that whatever happens with my illness, things will be okay	0	1	2	3	4

PART THREE

The Adult Hope Scale (AHS)

Directions: Read each item carefully. Using the scale shown below, please select the number that best describes YOU and put that number in the blank provided. 1. = Definitely False, 2. = Mostly False, 3. = Somewhat False, 4. = Slightly False, 5. = Slightly True, 6. = Somewhat True, 7. = Mostly True, 8. = Definitely True.

___ 1. I can think of many ways to get out of a jam.
___ 2. I energetically pursue my goals.
___ 3. I feel tired most of the time.
___ 4. There are lots of ways around any problem.
___ 5. I am easily downed in an argument.
___ 6. I can think of many ways to get the things in life that are important to me.
___ 7. I worry about my health.
___ 8. Even when others get discouraged, I know I can find a way to solve the problem.
___ 9. My past experiences have prepared me well for my future.
___ 10. I’ve been pretty successful in life.
___ 11. I usually find myself worrying about something.
___ 12. I meet the goals that I set for myself.

PART FOUR

Resilience Assessment Questionnaire-8 (RAQ-8)

Please answer the following questions by circling the relevant number.

1 = Never–5 = Always

1	I usually know how others perceive me	1	2	3	4	5
2	I am determined to achieve my lifetime ambitions	1	2	3	4	5
3	I can see my future clearly	1	2	3	4	5
4	I normally feel comfortable in new situations	1	2	3	4	5
5	I plan my next day in advance	1	2	3	4	5
6	I enjoy the challenge of unraveling puzzles and solving problems	1	2	3	4	5
7	In general, I like people	1	2	3	4	5
8	My most important relationships are my strongest	1	2	3	4	5

PART FIVE

Brief Resilience Scale (please respond to each item by marking one box per row)

		**Strongly agree**	**Disagree**	**neutral**	**agree**	**Strongly agree**
BRS1	I tend not to bounce back quickly after hard times	1	2	3	4	5
BRS2	I have a hard time making it through stressful events	5	4	3	2	1
BRS3	It takes me long to recover from a stressful event	1	2	3	4	5
BRS4	It is hard for me to snap back when something bad happens	5	4	3	2	1
BRS5	I usually come through difficult times with little trouble	1	2	3	4	5
BRS6	I tend to take a long time to get over setbacks in my life	5	4	3	2	1
